# Long-term visual outcomes of endophthalmitis and the role of systemic steroids in addition to intravitreal dexamethasone

**DOI:** 10.1186/s12886-020-01449-2

**Published:** 2020-05-06

**Authors:** Christopher D. Conrady, Richard M. Feist, Albert T. Vitale, Akbar Shakoor

**Affiliations:** 1 Department of Ophthalmology and Visual Sciences, John A. Moran Eye Center, University of Utah, Salt Lake City, UT USA; 2 Department of Ophthalmology and Visual Sciences, University of Michigan, 1000 Wall St, Ann Arbor, MI 48103 USA

**Keywords:** Endophthalmitis, Steroids, Post-procedure, Infection

## Abstract

**Background:**

The purpose of this study was to evaluate the role of systemic steroids in post-procedural endophthalmitis as the role of intravitreal steroids in treatment algorithms of endophthalmitis remain controversial.

**Methods:**

This is a retrospective analysis from a single tertiary referral center of all patients older than 18 years old that developed presumed post-procedure endophthalmitis and were treated at our center from 2009 to 2018.

**Results:**

Eighty-three patients were followed after being treated for post-procedural endophthalmitis that either received systemic steroids or did not around the time of diagnosis. Almost 30 % of all patients regained a final visual acuity of 20/40 or better, while 31.2% had poor visual outcomes of count fingers or worse. Non-clearing debris was the most significant long-term complication. Visual improvement plateaued in 67.7% by 1 month after diagnosis and initial treatment in both groups. There was no difference in visual outcomes when comparing the sixteen patients that received systemic steroids and the sixty-seven that did not; however, no enucleation or evisceration was required in patients receiving systemic steroids. Five patients that did not receive systemic steroids required an enucleation or evisceration due to a blind, painful eye.

**Conclusions:**

The use of systemic steroids does not seem to worsen long-term outcomes of endophthalmitis compared to those patients that did not receive them and they may prove beneficial in the most severe cases by reducing the risk of losing the globe altogether.

## Background

Endophthalmitis is a rare but significant vision-threatening risk of any intraocular procedure. In fact, approximately 40% of endophthalmitis cases associated with cataract surgery and greater than 90% of those associated with trabeculectomy surgeries will develop visual acuities worse than 20/200. These post-operative infections result in an estimated 83% rise in overall medical costs alone [1–3]. Given the significant ocular morbidity associated with intraocular infections, and despite the introduction of systemic antibiotics with better ocular penetrance (i.e.fluoroquinolones), and the use of smaller vitrectomy port sizes, the Endophthalmitis Vitrectomy Study (EVS) published in 1995 remains the foundation of current treatment algorithms [4]. Due to the lack of more recent randomized controlled trials and significant exclusion criteria in the EVS such as the exclusion of non-cataract-associated procedures, cases with severe intraocular inflammation, and indolent courses, there is no consensus as to the management of post-procedure endophthalmitis [5]. This has become increasingly apparent with the advent and expansion of intravitreal injections for multiple chorioretinal diseases since the EVS was conducted [5, 6]. Despite a lack of randomized trials, groups have questioned the applicability of the EVS recommendation of performing vitrectomies only in those eyes with light perception or worse visual acuities; however, data has been mixed from these studies [7–9]. Recent attention has been directed towards prevention strategies and early detection of post-operative endophthalmitis rather than management to improve long-term outcomes once an infection has manifested [5, 10].

In the following study, we aimed to assess outcomes of all post-procedure endophthalmitis cases treated at our institute with a special attention to those that received steroids during their treatment. In a recent, randomized control trial evaluating the utility of intravitreal dexamethasone in bacterial endophthalmitis, there was no difference in visual outcomes in those given intravitreal steroid compared to placebo [11]. We hypothesized that the use of a larger dose and more prolonged tapers of systemic steroids, such as those employed in other infectious uveitides such as toxoplasma retinochoroiditis and acute retinal necrosis, would promote resolution of the severe intraocular inflammation seen in endophthalmitis resulting in better long-term outcomes.

## Methods

### Human subject data collection

The study was approved by the Institutional Review Board at the University of Utah (Protocol 00100987) and the research presented adhered to the tenets of the Declaration of Helsinki. All subjects (> 18 years old) were identified by retrospective screening of the electronic medical record of patients seen and treated for any post-procedure related endophthalmitis at the John A. Moran Eye Center, a tertiary referral center. Endophthalmitis was considered the primary diagnosis when there was significant intraocular inflammation that included both the aqueous and vitreous cavities and there was a known history of an intraocular intervention. Treatment, surgery details, and subsequent follow up were then collected from retrospective chart review. Data (visual acuities, inciting procedure and organism, use of oral steroids, long term outcomes) was then compiled in REDCap [12]. The only patients excluded were those that developed endogenous endophthalmitis.

Nearly all patients (> 90%) received intravitreal antibiotics (vancomycin 1 mg/0.1 mL; ceftazidime 2.25 mg / 0.1 mL) and intravitreal dexamethasone (400 μg / 0.1 mL) at time of intervention, and either a tap and inject or vitrectomy at time of diagnosis based off of EVS criteria. Approximately 40% of patients also received a course of oral fluoroquinolone at time of diagnosis (37% of patients not receiving oral steroids and 56% of those receiving them). Patients that received oral steroids were given a 1 mg/kg starting dose as seen in many uveitis practices within 24–48 h of intravitreal antibiotic administration to quell intraocular inflammation and a taper of 60 mg, 40 mg, and then 20 mg, with dose reduction every 3–5 days was followed depending on clinical response and the clinician’s discretion. All anterior chamber inflammation was treated with topical steroids and drops tapered with clinical improvement.

Statistical analysis was performed using chi squared analysis or student *t* test. A *p* value less than 0.05 was considered statistically significant.

## Results

**Fig. 1:**
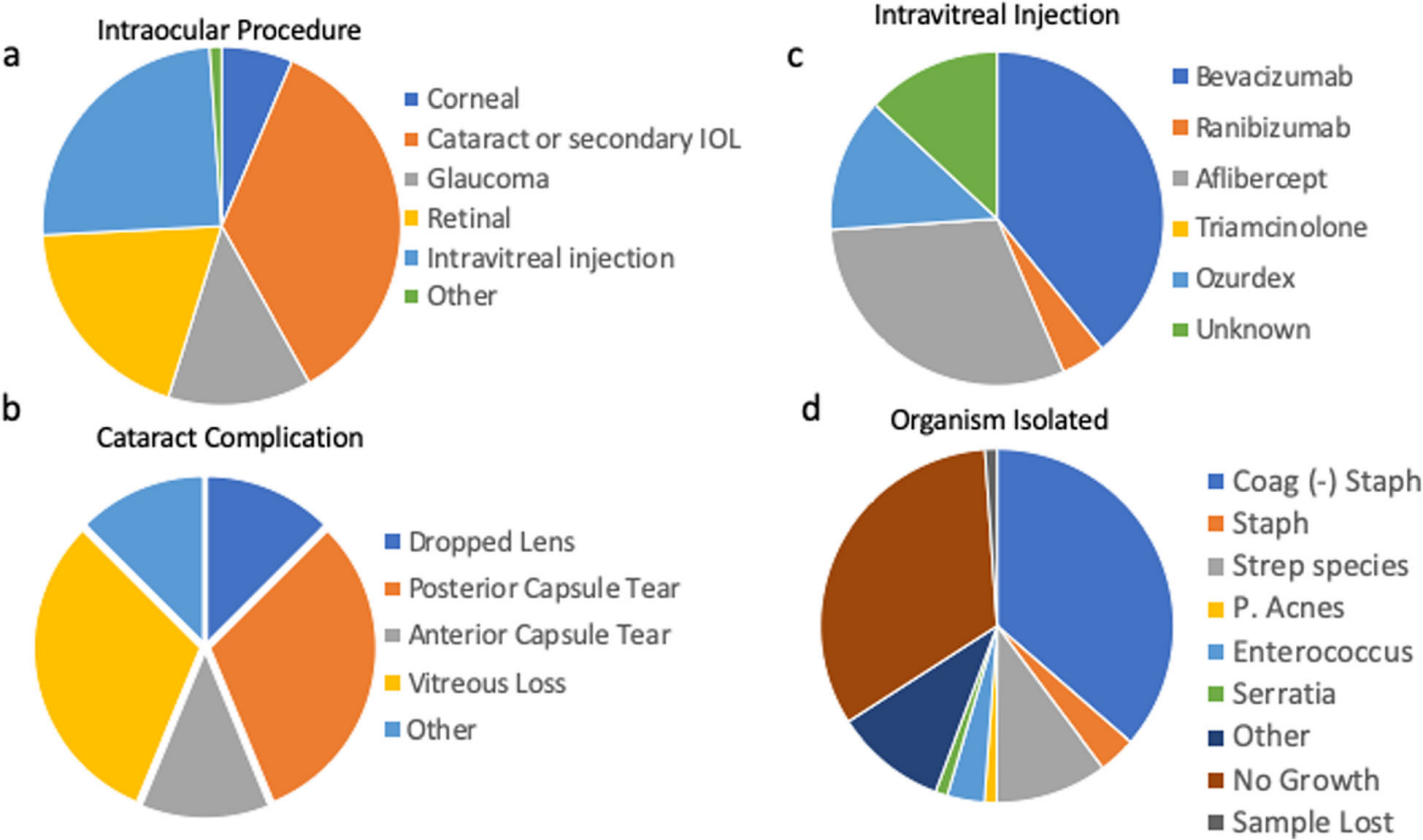
Inciting procedures and resultant organisms. The type of intraocular procedure (**a**), specific intravitreal injection (**b**), intraoperative cataract complications (**c**), and organism isolated (**d)** are seen. Coag, coagulase; IOL, intraocular lens; P. acnes, *Propionibacterium acnes*

Eighty-three patients who developed and were treated for endophthalmitis following an intraocular procedure were identified by retrospective chart review from 2009 to 2018 at the John A. Moran Eye Center. Patients were on average 72.1 years old (range 19–94), non-diabetic (63.4%), female (63.9%), and followed for 471 days (Table 1). The most frequent inciting interventions included complicated cataract or secondary IOL placement (39.7%), intravitreal injections (27.7%), and retinal surgeries (21.7%) (Table 1, Fig. 1a-c). More frequently utilized intravitreal medications at our facility were associated with more total cases of post-intravitreal injection endophthalmitis (Fig. 1b, data not shown). Most patients received intravitreal vancomycin, ceftazidime, and dexamethasone and underwent a vitreous tap with the sample analyzed for microbial organisms at the time of diagnosis, while 12% underwent a primary diagnostic vitrectomy with concomitant intravitreal vancomycin, ceftazidime, and dexamethasone (Table 1). Approximately a third of patients required additional treatment, either a pars plana vitrectomy (PPV) and/or intravitreal antibiotics and nearly half of secondary interventions were PPV with injection of intravitreal antibiotics (Table 1). Fortunately, only 12.2% of patients required a third intervention, additional intravitreal antibiotics with or without a concurrent PPV, to control the ongoing infection (Table 1).

**Table 1:** Demographics of patients included in the study

Demographics	Mean	Range
**Age (years)**	72.1	19–94
**Sex**		**Total # (n)**
Male		30
Female		53
Total		83
	**%**	
**Diabetic**		
Yes	28	
No	63.4	
Unkown	8.5	
**Lens Status at Diagnosis**		
Phakic	19.3	
Pseudophakic	77.1	
Aphakic	3.6	
**Lens Status at final evaluation**		
Phakic	7.4	
Pseudophakic	86.4	
Aphakic	6.2	
**Surgical Type**		
Corneal	7.2	
Cataract or secondary IOL	39.7	
Glaucoma	14.5	
Retinal	21.7	
Intravitreal injection	27.7	
Other	1.2	
**Initial Treatment**		
Tap and Inject	88	
Vitrectomy	12	
**Retreatment Needed**	33.7	
**3rd Treatment Needed**	12.2	
**Systemic Steroids**	19.3	
		**Mean**
**Follow up (days)**	1 to 2938 d	471.81

*d* days, *IOL* intraocular lens, *n* number

In each case, the vitreous specimen was sent for typical microbiological analysis (standard plating protocols). Similar to results found at other institutes and the EVS, 35.8% of specimens could not identify a pathogen and the most common organism present were coagulase negatives staphylococcus strains (Fig. 1d) [6, 13]. This was followed by Streptococcus species (Fig. 1d). Interestingly, there were 10 pathogens identified that were resistant to levofloxacin (data not shown).

**Fig. 2:**
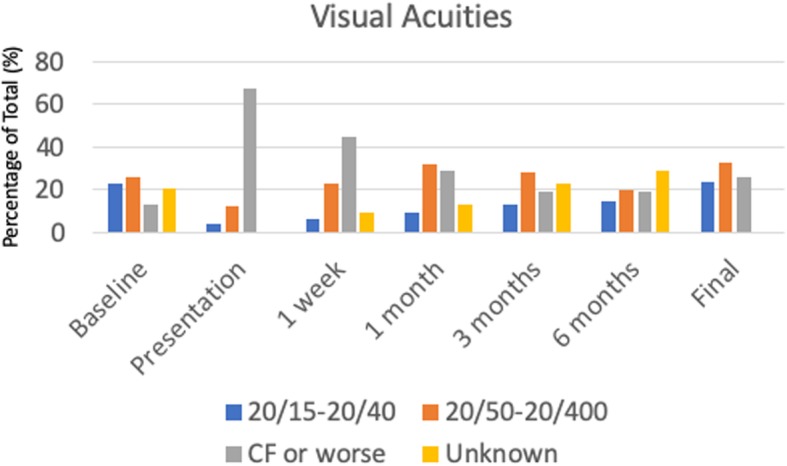
Visual acuities of entire cohort. The visual acuities were collected and grouped into 20/20–20/40, 20/50–20/400, count fingers or worse. They are expressed in percentages of the entire cohort CF, count fingers.

**Fig. 3:**
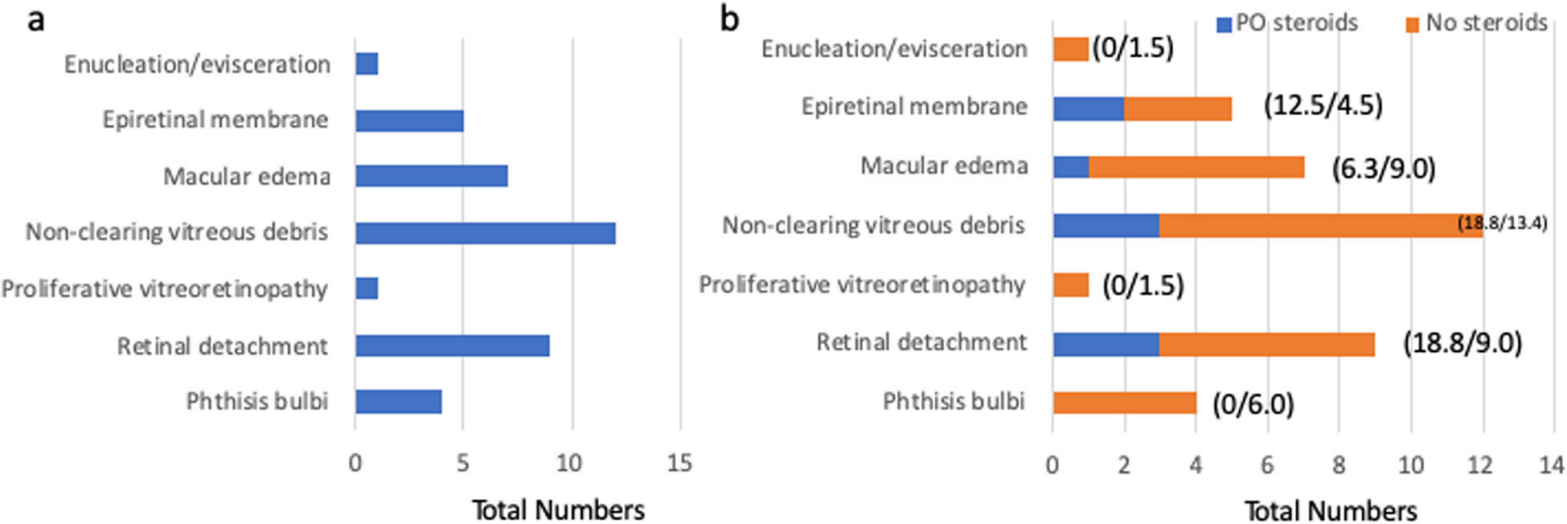
Long-term complications of endophthalmitis. (**a**) The overall total number of complications can be seen. (**b**) The complications were then separated into those that received systemic steroids (blue bar) and those that did not (orange). Percentage of the total group is found in the parentheses as follows: (oral steroids / no steroids). PO, oral

Analysis of the long-term outcomes of the patients in our study revealed that almost 30 % of patients regained a final visual acuity of 20/40 or better, while 31.2% had poor visual outcomes of count fingers or worse (Fig. 2). There was an interesting trend in which 67.7% of patients followed for at least 90 days had visual acuities at 1 month that were within 3 lines of their final visual acuity (Fig. 2). Thirty eyes (36.1%) had long term complications from endophthalmitis. The most common complications included non-clearing vitreous debris, retinal detachments, macular edema, and epiretinal membranes (Fig. 3a). Furthermore, for those patients that had not had cataract prior to developing endophthalmitis, most would require cataract surgery during follow up as only 7.4% of patients were phakic at last follow up compared to 19.3% prior to the infection (Table 1).

**Fig. 4:**
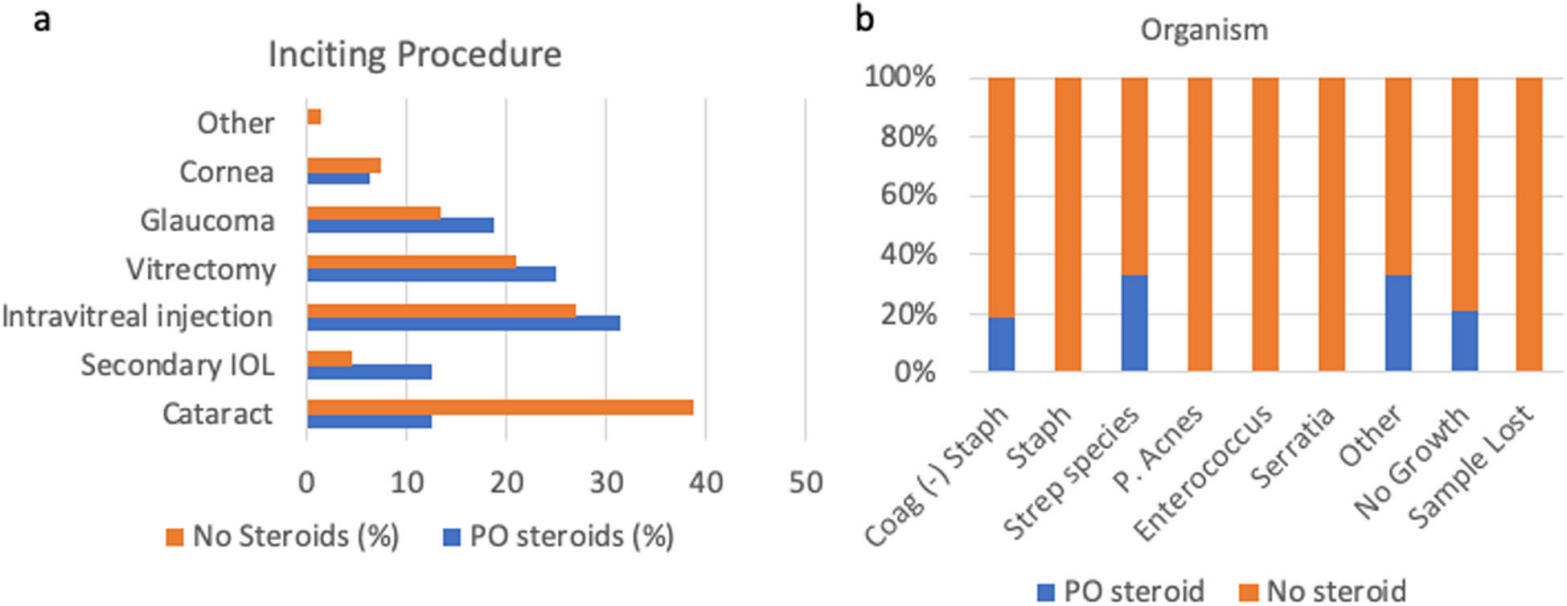
Inciting procedures and organisms in patients that received systemic steroids on those that did not. (**a**-**b**) Percentage of the total number of surgical cases (**a**) and organism isolated (**b**) of the systemic steroid receiving group (blue bar) versus those patients that did not receive systemic steroids (orange bar) respectively. IOL, intraocular lens; P. acnes, *Propionibacterium acnes*; PO, oral

**Fig. 5:**
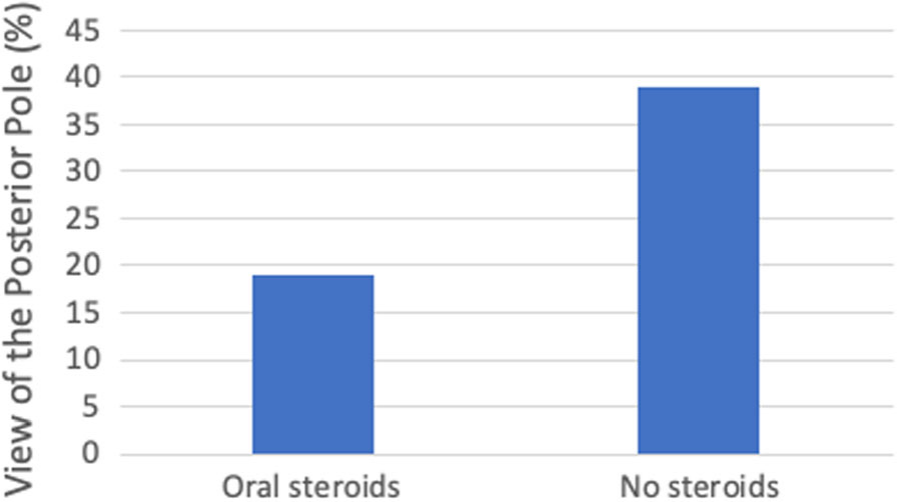
Visualization of the Posterior Pole. Visualization of the posterior segment at time of endophthalmitis diagnosis was compared between the group receiving systemic steroids and those that did not. Data plotted as percentage

The role of systemic steroids in endophthalmitis was then analyzed as prior studies have only evaluated the efficacy of intravitreal dexamethasone and found no substantial effect on visual outcomes [11, 14, 15]. Nineteen percent of the patients (*n* = 16) included in this study received an oral steroid taper consisting of 1 mg/kg body weight prednisone that was started 24–48 h after intravitreal antibiotics and dexamethasone. Dose was tapered every depending on clinical response (Table 1). Patients who received steroids were on average 69.3 ± 14.0 years old and predominately male (68.8%) compared to those that did not who were 72.7 ± 13.1 years old and predominately female (71.6%) [Table 2]. Glaucoma procedures and secondary intraocular lenses implantation constituted more inciting interventions in the group receiving systemic steroids, while post-cataract cases represented more overall cases in the group not receiving steroids (Fig. 4a). Both groups underwent similar primary interventions with 88.1% in the systemic steroid-free and 87.5% of the steroid-receiving groups undergoing a vitreous paracentesis and injection of antibiotics and dexamethasone (Table 2). Isolated organisms in the group receiving systemic steroids were mostly Strep species or coagulase negative staphylococcus species (Fig. 4b). The patients receiving systemic steroids had more severe inflammation at diagnosis than those that did not receive steroids as seen by the inability to examine the posterior segment due to significant vitreous inflammatory debris (Fig. 5, *p* = 0.1340).

**Table 2:** Comparison of those patients receiving systemic steroids and those that did not

	No steroids	Systemic steroids
Age (years)	72.7	69.3
	**%**	**%**
Male	28.4	68.8
Female	71.6	31.3
Diabetic	18	25
Average follow up (days)	477	449

*PO* oral

**Fig. 6:**
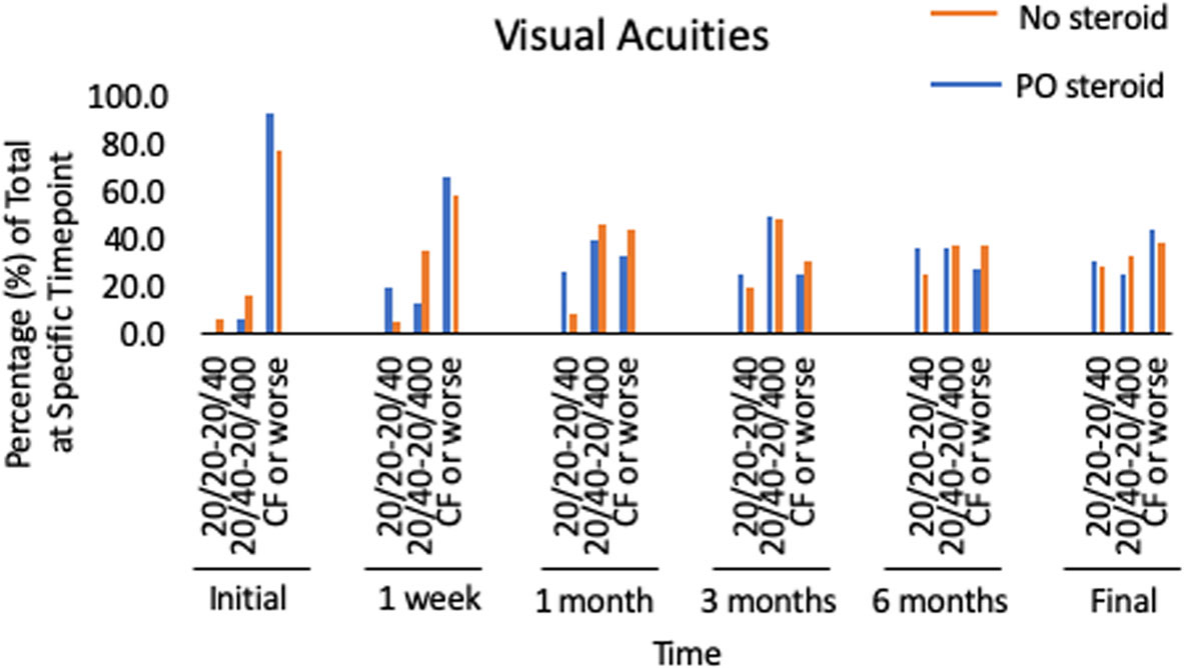
Comparing visual acuities of those patients receiving systemic steroids and those that did not. The visual acuities were collected and grouped into 20/20–20/40, 20/50–20/400, count fingers or worse. They are expressed in percentages of their respective cohort at the indicated time point. The orange bar represents those that did not receive systemic steroids and the blue bars those did. CF, count fingers; PO, oral

Final visual acuity outcomes were indistinguishable when comparing the two groups (Fig. 6). In the group receiving systemic steroids, however, no patient developed a phthsical and/or blind, painful eye throughout follow up, while patients in the group that did not receive steroids were less fortunate with no patient undergoing an enucleation/evisceration within 90 days of inciting event (*p* = 0.26, Fig. 3b). Furthermore, overall complication rates were similar amongst the two groups (systemic steroids: 37.5% vs. no steroids: 35.8%) suggesting that systemic steroids do not put patients at higher risk of developing long-term complications as one might expect with immune altering drugs during active infections (Fig. 3b). These two findings were especially striking as the patients receiving systemic steroids had worse presenting visual acuities on average than controls (Fig. 6). Again, as seen in our entire cohort earlier, vision improvement stabilized by 1 month (Figs. 2, 6).

## Discussion

Despite advances in surgical techniques, diagnostic tools, and the availability of new antibiotics, poor outcomes are still common following post-procedure endophthalmitis and certain interventions, such as trabeculectomies are more prone to exceptionally poor prognoses [5]. Systemic steroids may help to salvage and/or preserve eyes with an exceptionally poor prognosis as seen in this study. While not statistically significant in this study when comparing outcomes of patients receiving systemic steroids and those that did not, there was a trend (*p* = 0.26); however, the study was too small to confirm this. If extrapolated from the group that did not receive systemic steroids, though, at least one patient in the systemic steroid group should have lost their eye (1 in 13.4). It is important to emphasize that systemic steroids did not increase the risk of complications stemming from the infection as the pathogen was under adequate antimicrobial therapy at the time systemic steroids were commenced in our study as unopposed steroids during endophthalmitis can lead to significant worsening of the infection and inflammation and even loss of the eye [16]. After enucleation or evisceration, patients have a worse quality of life and nearly 40% of them will stop participating in leisure activities in one study. [17] Rates of depression, anxiety, and problems with appearance are also higher in patients that have undergone enucleation than the general public [18, 19]. As such, preservation of the eye has psychosocial health benefits for the patient even when vision has been significantly compromised. The use of systemic steroids should at least be considered in the worst endophthalmitis cases and those cases known to have historically poor prognoses such as post-trabeculectomy intraocular infections to help preserve the globe if nothing else [5].

Higher concentrations of specific cytokines analyzed in post-operative endophthalmitis were associated with poorer visual outcomes [20]. In our study there was a selection bias towards prescribing systemic steroids to the most severe cases (worse presenting visual acuities, glaucoma procedures, more severe inflammation as manifested by lower rates of visualization of the posterior pole, and Strep species) [5]. It is these cases that have the worst visual outcomes and will likely have the most benefit from earlier inflammatory control due to the role of inflammation in worsening retinal pathology. Animal data has suggested intraocular inflammation contributes to retinal damage leading groups to propose augmenting the immune system with corticosteroids during endophthalmitis to limit irreversible retinal damage and we hypothesize that this inflammatory event drives the high complication rate we found in our patient population [21]. Presumably, patients with less severe presenting features may also benefit from earlier inflammatory control due to this as well and would benefit from systemic steroids. While several studies have evaluated the efficacy of intravitreal dexamethasone at the time of diagnosis and treatment, the results have been mixed with the most recent randomized, control trial finding no benefit on visual outcomes [11, 15, 22]. Unfortunately, the half-life of intravitreal dexamethasone is short, approximately 5.5 h, and is unlikely to have any meaningful, long-lasting effect on an ongoing, severe inflammatory event such as endophthalmitis [14]. Animal models of endophthalmitis have shown that the proinflammatory cytokines peak within 12 h after inoculation but remain significantly elevated for 7 days [23]. This coincided with clinical examination in which signs of infection could be detected for up to 7 days after inoculation in this same study [23]. These laboratory findings would support our initial hypothesis that intravitreal dexamethasone is unlikely to have any meaningful effect on a process that lasts days to weeks. On the other hand, a prolonged systemic steroid taper similar to that seen in the management of other infectious uveitides with intense inflammatory responses (i.e. toxoplasmic chorioretinitis and acute retinal necrosis) would be more appropriate and by hastening inflammatory control, provide better visualization for subsequent examinations, control symptoms, and theoretically lessen pathologic ocular structural complications [24, 25].

There are several other important findings that can be taken from this study. First, one-month visual acuities were within three lines of the final visual acuity. Although not completely unexpected as active inflammation has typically resolved within the first few weeks following diagnosis and treatment, [23] this finding is most important from a prognostic standpoint to help prepare patients for their new future. Certain clinical features such as a lack of cataract surgery complications, better presenting visual acuities, and an ability to visualize the posterior segment portend a better visual prognosis than the converse or when corneal edema or a virulent organism is identified [26, 27]. These features may not modify the visual prognosis in a patient receiving anti-VEGF therapy for macular degeneration with concomitant macular pathology prior to developing endophthalmitis. However, visual acuities at one-month might predict significant, underlying visually-inhibiting ocular pathology in the specific patient in question.

This report has several limitations. A retrospective case series of patients with endophthalmitis does not have the same power of a randomized, controlled trial may have such as the EVS [6]. This study was also underpowered to answer the underlying question of whether systemic steroids are of benefit in overall treatment of endophthalmitis. Our study suggests that while there was no benefit with respect to final visual outcome, the use of systemic corticosteroids may reduce ocular structural damage and rates of unsalvageable globes that ultimately become phthisical and/or require evisceration or enucleation.

Fortunately, post-procedure endophthalmitis is not a common occurrence with rates as low as 0.053–0.09% with the institution of perioperative antibiotic prophylaxis following cataract surgery and rates following intravitreal injection between 0.004–0.038% [28–32]. As such, a large, multicenter study would need to be pursued to evaluate the efficacy of systemic steroids. Hopefully smaller, retrospective studies such as this one become the foundation of support needed to garner larger financial commitments from funding agencies going forward to help better understand the best management strategy for endophthalmitis and help clarify some of the questions left unanswered by the EVS [4, 6].

## Conclusions

The use of systemic steroids does not seem to worsen long-term outcomes of endophthalmitis and may prove beneficial in the most severe cases. As such, systemic steroids should be considered in the treatment of endophthalmitis but only after the infection has been adequately addressed.

## Data Availability

All data will be made readily available upon request.

## Abbreviations

EVS: Endophthalmitis Vitrectomy Study
mg: Milligram
PPV: Pars plana vitrectomy
VEGF: Vascular endothelial growth factor

## References

[1] Schmier JK, Hulme-Lowe CK, Covert DW, Lau EC (2016). An updated estimate of costs of endophthalmitis following cataract surgery among Medicare patients: 2010-2014. Clin Ophthalmol Auckl NZ.

[2] Poulsen EJ, Allingham RR (2000). Characteristics and risk factors of infections after glaucoma filtering surgery. J Glaucoma.

[3] Friling E, Lundström M, Stenevi U, Montan P (2013). Six-year incidence of endophthalmitis after cataract surgery: Swedish national study. J Cataract Refract Surg.

[4] Grzybowski A, Turczynowska M, Kuhn F (2018). The treatment of postoperative endophthalmitis: should we still follow the endophthalmitis vitrectomy study more than two decades after its publication?. Acta Ophthalmol.

[5] Brundrett A, Conrady CD, Shakoor A, Lin A (2018). Current strategies for prevention and treatment of postoperative Endophthalmitis. Curr Ophthalmol Rep.

[6] Results of the Endophthalmitis Vitrectomy Study. A randomized trial of immediate vitrectomy and of intravenous antibiotics for the treatment of postoperative bacterial endophthalmitis. Endophthalmitis Vitrectomy Study Group. Arch Ophthalmol. 1995;113(12):1479–96. 7487614

[7] Ho I-V, Fernandez-Sanz G, Levasseur S (2019). Early pars Plana Vitrectomy for treatment of acute infective Endophthalmitis. Asia-Pac J Ophthalmol Phila Pa.

[8] Kurniawan ED, Rocke JR, Sandhu SS, Allen PJ (2018). Predictors of visual outcome and the role of early vitrectomy in streptococcal endophthalmitis. Clin Exp Ophthalmol.

[9] Shalaby A, Cherubini SDS, Lockwood A, Newsom R. Postoperative Endophthalmitis: incidence, causes and comparison between medical and surgical treatment in a United Kingdom region in the last 10 years. Acta Ophthalmol (Copenh) 93. 2015. 10.1111/j.1755-3768.2015.0673.

[10] Conrady CD, Feistmann JA, Roller AB, et al. Hemorrhagic vasculitis and retinopathy heralding as an early sign of bacterial endophthalmitis after intravitreal injection. Retin Cases Brief Rep. 2017. 10.1097/ICB.0000000000000601. 10.1097/ICB.0000000000000601 28594738

[11] Manning S, Ugahary LC, Lindstedt EW (2018). A prospective multicentre randomized placebo-controlled superiority trial in patients with suspected bacterial endophthalmitis after cataract surgery on the adjuvant use of intravitreal dexamethasone to intravitreal antibiotics. Acta Ophthalmol.

[12] Harris PA, Taylor R, Thielke R (2009). Research electronic data capture (REDCap) - a metadata-driven methodology and workflow process for providing translational research informatics support. J Biomed Inform.

[13] Doan T, Acharya NR, Pinsky BA (2017). Metagenomic DNA sequencing for the diagnosis of intraocular infections. Ophthalmology.

[14] Gan IM, Ugahary LC, van Dissel JT, van Meurs JC (2005). Effect of intravitreal dexamethasone on vitreous vancomycin concentrations in patients with suspected postoperative bacterial endophthalmitis. Graefes Arch Clin Exp Ophthalmol Albrecht Von Graefes Arch Klin Exp Ophthalmol.

[15] Albrecht E, Richards JC, Pollock T (2011). Adjunctive use of intravitreal dexamethasone in presumed bacterial endophthalmitis: a randomised trial. Br J Ophthalmol.

[16] Bucher RS, Hall E, Reed DM (1960). (2005) effect of intravitreal triamcinolone acetonide on susceptibility to experimental bacterial endophthalmitis and subsequent response to treatment. Arch Ophthalmol Chic Ill.

[17] Rasmussen MLR (2010). The eye amputated – consequences of eye amputation with emphasis on clinical aspects, phantom eye syndrome and quality of life. Acta Ophthalmol.

[18] Ye J, Lou L, Jin K, et al. Vision-Related Quality of Life and Appearance Concerns Are Associated with Anxiety and Depression after Eye Enucleation: A Cross-Sectional Study. PLoS ONE. 2015:10. 10.1371/journal.pone.0136460. 10.1371/journal.pone.0136460 PMC4552790 26317860

[19] Brandberg Y, Kock E, Oskar K (2000). Psychological reactions and quality of life in patients with posterior uveal melanoma treated with ruthenium plaque therapy or enucleation: a one year follow-up study. Eye.

[20] Sauer A, Ermanno C, Gaucher D (2017). Intraocular cytokine levels in post-cataract Endophthalmitis and their association with visual outcome. Ocul Immunol Inflamm.

[21] Novosad BD, Astley RA, Callegan MC (2011). Role of toll-like receptor (TLR) 2 in experimental Bacillus cereus endophthalmitis. PLoS One.

[22] Gan IM, Ugahary LC, van Dissel JT (2005). Intravitreal dexamethasone as adjuvant in the treatment of postoperative endophthalmitis: a prospective randomized trial. Graefes Arch Clin Exp Ophthalmol Albrecht Von Graefes Arch Klin Exp Ophthalmol.

[23] Petropoulos IK, Vantzou CV, Lamari FN (2006). Expression of TNF-α, IL-1β, and IFN-γ in Staphylococcus epidermidis slime-positive experimental endophthalmitis is closely related to clinical inflammatory scores. Graefes Arch Clin Exp Ophthalmol.

[24] Harrell M, Carvounis PE (2014). Current treatment of toxoplasma Retinochoroiditis: an evidence-based review. In : J. Ophtalmol.

[25] Shantha JG, Weissman HM, Debiec MR (2015). Advances in the Management of Acute Retinal Necrosis. Int Ophthalmol Clin.

[26] Combey de Lambert A, Campolmi N, Cornut P-L (2013). Baseline factors predictive of visual prognosis in acute postoperative bacterial endophthalmitis in patients undergoing cataract surgery. JAMA Ophthalmol.

[27] Yospaiboon Y, Intarapanich A, Laovirojjanakul W (2018). Factors affecting visual outcomes after treatment of infectious endophthalmitis in northeastern Thailand. Clin Ophthalmol Auckl NZ.

[28] Moshirfar M, Feiz V, Vitale AT (2007). Endophthalmitis after uncomplicated cataract surgery with the use of fourth-generation fluoroquinolones: a retrospective observational case series. Ophthalmology.

[29] Ravindran RD, Venkatesh R, Chang DF (2009). Incidence of post-cataract endophthalmitis at Aravind eye hospital: outcomes of more than 42,000 consecutive cases using standardized sterilization and prophylaxis protocols. J Cataract Refract Surg.

[30] Stem MS, Rao P, Lee IJ (2019). Predictors of Endophthalmitis after Intravitreal injection: a multivariable analysis based on injection protocol and Povidone iodine strength. Ophthalmol Retina.

[31] Dossarps D, Bron AM, Koehrer P (2015). Endophthalmitis after Intravitreal injections: incidence, presentation, management, and visual outcome. Am J Ophthalmol.

[32] Al-Rashaed S, Alsulaiman SM, Alrushood AA (2016). Incidence of Endophthalmitis after Intravitreal anti-vascular endothelial growth factor: experience in Saudi Arabia. Middle East Afr J Ophthalmol.

